# BCG immunotherapy promotes tumor-derived T-cell activation through the FLT3/FLT3LG pathway in bladder cancer

**DOI:** 10.7150/jca.90085

**Published:** 2024-01-01

**Authors:** Wei Zhang, Lu Yu, Zhiguang Chang, Haiyun Xiong

**Affiliations:** 1 Department of Urology, The Seventh Affiliated Hospital, Sun Yat-Sen University, Shenzhen, China; 2 Emergency and Disaster Medical Center, The Seventh Affiliated Hospital, Sun Yat-Sen University, Shenzhen, China; 3 Edmond H. Fischer Translational Medical Research Laboratory, Scientific Research Center, The Seventh Affiliated Hospital, Sun Yat-Sen University, Shenzhen, China; 4 Clinical laboratory, The Seventh Affiliated Hospital, Sun Yat-Sen University, Shenzhen, China

**Keywords:** Bacillus Calmette-Guérin (BCG), FLT3, FLT3LG, T-cell activation

## Abstract

Bladder instillation therapy is a common treatment for superficial or nonmuscle invasive bladder cancer. After surgery or reresection, chemotherapy drugs (epirubicin) or medications such as Bacillus Calmette-Guérin (BCG) are used for bladder instillation therapy, which can reduce the risk of bladder cancer recurrence and progression. However, the specific mechanism by which BCG stimulates the antitumor response has not been thoroughly elucidated. Additionally, although BCG immunotherapy is effective, it is difficult to predict which patients will have a positive response. In this study, we explored the BCG-induced immune response and found that high levels of Fms-related receptor tyrosine kinase 3 ligand (FLT3LG) were expressed after BCG treatment. This FLT3LG can directly act on CD8^+^ T cells and promote their proliferation and activation. The use of FLT3 inhibitors can neutralize the antitumor effects of BCG. In vitro experiments showed that FLT3LG can synergize with T-cell receptor activators to promote the activation of tumor-derived T cells. This study partially elucidates the mechanism of CD8^+^ T-cell activation in BCG immunotherapy and provides a theoretical basis for optimizing BCG instillation therapy in bladder cancer

## Introduction

Bladder cancer is the ninth most common malignant tumor worldwide^1^. It predominantly affects males, with approximately 75% of patients being male^2^. Approximately 80% of bladder cancer patients present with nonmuscle invasive bladder cancer (NMIBC). The treatment for NMIBC includes transurethral resection of the tumor, followed by bladder instillation therapy based on grading and other risk factors, such as tumor size or multifocality, to reduce the risk of recurrence or progression. The use of intravesical immunotherapy with Bacillus Calmette-Guérin (BCG), a live attenuated strain of Mycobacterium bovis used for tuberculosis vaccination, was first described in humans by Morales et al. in 1976^3^. BCG immunotherapy is considered the gold standard adjuvant therapy for NMIBC with high progression risk (i.e., T1 stage tumors, high-grade cancers, carcinoma in situ, and multifocal and recurrent Ta stage tumors >3 cm), and it is also recommended for intermediate-risk NMIBC^4^. The mechanisms underlying tumor immunity mediated by BCG immunotherapy have been extensively studied but are not fully understood. Preclinical and clinical research has shown that a strong local inflammatory response to BCG involves several steps^5^: 1) BCG attachment to the urothelium, 2) BCG internalization, 3) induction of the innate immune response, and 4) induction of adaptive immunity.

BCG immunotherapy induces a local innate immune response, including neutrophils, macrophages, and NK cells^6^, ^7^. Recent reports have shown that NK cells can secrete FLT3LG to exert antitumor activity^8^. As effector cells against tumor cells, cytotoxic CD8 T cells have also been confirmed to be essential for the effectiveness of BCG immunotherapy. In a mouse bladder cancer tumor model, the antitumor effects of BCG immunotherapy were lost when CD4^+^ or CD8^+^ T cells were depleted, indicating their crucial role in the induced antitumor activity of BCG^9^. Increased tumor-infiltrating CD4^+^ T-cell counts and an increased CD4^+^:CD8^+^ T-cell ratio were significantly correlated with improved patient response to BCG in bladder cancer patients' tumor samples^10^. However, the underlying mechanisms of CD8 T-cell activation within the bladder are still not clear.

Here, we propose the hypothesis that during BCG immunotherapy for bladder cancer, immune activation can lead to a significant amount of FLT3LG secretion, which in turn activates cytotoxic CD8^+^ T cells within the tumor to exert antitumor activity. Our results demonstrate that after BCG treatment, urinary FLT3LG levels are significantly increased compared to other instillation methods, and FLT3LG can assist in the in vitro activation of CD8^+^ T cells. This study highlights the critical role of FLT3/FLT3LG in BCG immunotherapy for bladder cancer and provides novel candidate targets for optimizing BCG immunotherapy in future studies.

## Materials and Methods

### Patient samples

To analyze FLT3LG levels in bladder instillation therapy patients enrolled in The Seventh Affiliated Hospital, Sun Yat-Sen University. Informed consent was obtained from all study subjects prior to their enrollment in this study. The patients was selected with T1 stage tumors or carcinoma in situ (CIS) according to Association of Urology Guidelines^11^. Sample FLT3LG levels were measured 24 hours after BCG bladder instillation. The sample collection for this study was reviewed and approved by the Ethics Committee of The Seventh Affiliated Hospital, Sun Yat-Sen University (Approved number: KY-2020-028-01). FLT3LG was detected using a Human Flt-3 Ligand/FLT3L Quantikine ELISA Kit (DFK00, R&D) according to the manufacturer's instructions.

### Survival rate analysis

FLT3LG expression and BLCA survival data were downloaded from the TCGA database (http://cancergenome.nih.gov/). The “ComplexHeatmap” function in R was used to generate the oncopPrint graph. FLT3LG mRNA expression in cancer and adjacent normal tissues was compared, and patients were subsequently divided into high expression and low-expression groups using the median as the cutoff value. The correlation between mRNA expression (high or low) and overall survival (OS) was analyzed by Kaplan-Meier analysis and log-rank test. R was used to generate OS and disease/progression-free survival Kaplan-Meier curves.

### Tumor model and CD8^+^ T-cell isolation and treatment

The MB49 cell line was purchased from ATCC and maintained in in vitro culture (DMEM, 10% FCS, and 1% penicillin/streptomycin at 37°C and 5% CO_2_). Six- to eight-week-old C57BL/6 mice (weight range, 18-20 g) were purchased from GemPharmatech Co., Ltd. The study protocols were approved by the Institutional Animal Care and Use Committee, Sun Yat-Sen University. To construct the subcutaneous tumor model, each mouse was shaved and subsequently inoculated with 10^6^ CT26 cancer cells subcutaneously. Mice were sacrificed when the tumor volumes reached approximately 500 mM^3^ (tumor volume = 1/2 × a × b^2^; a = length and b = width), and tumor tissues were collected for subsequent experiments. FLT3LG was detected using a Mouse/Rat Flt-3 Ligand/FLT3L Quantikine ELISA Kit (MFK00, R&D) according to the manufacturer's instructions.

Mouse tumor tissues were homogenized using a rubber tip and 70 μm cell strainer. CD8^+^ T cells were isolated from single-cell suspensions using a CD8^+^ T-Cell Isolation Kit (Miltenyi, 130-090-859). Purified CD8^+^ T cells were identified using flow cytometry, as >90% of isolated cells were CD3^+^CD8^+^ phenotypes. Purified cells (5 × 10^4^) were placed in the well plate, and anti-mouse CD3ε, clone 145-2C11 (100339, Biolegend) or/and 100 ng/mL FLT3LG were added to stimulate for 12 hours. Cells were washed and collected for RNA extraction.

### Estimation of STromal and Immune cells in MAlignant Tumor tissues using Expression data (ESTIMATE)

ESTIMATE is a comprehensive resource for analyzing stromal and immune cell infiltration in tumors. Tumor purity was estimated by a previously reported method^12^ using the TCGA gene expression profile to infer immune and stromal scores. Immune and stromal scores represented immune and stromal cell infiltration, respectively. A combined ESTIMATE of these two scores was evaluated.

### Correlation between FLT3LG and immune infiltration and TME

Functional enrichment analysis results from a previous study suggest that FLT3LG family members play a role in immunotherapy-related signaling pathways. To further validate this finding, single-sample gene set enrichment analysis (ssGSEA) was used to determine the involvement of FLT3LG in the infiltration of 24 immune cells using a previously reported method^13^. Subsequently, the association between the FLT3LG gene and immune cells was assessed in bladder urothelial carcinoma (BLCA). The 24 kinds of tumor immune cell infiltration data were obtained from the ImmunCellAl database (http://bioinfo.life.hust.edu.cn/ImmuCellAI#!/). Correlation analysis between the tumor microenvironment and FLT3LG was performed using the method described in a previous article^13^. Heatmaps were used to show the correlation between FLT3/FLT3LG and TME pathway scores.

### Kyoto Encyclopedia of Genes and Genomes (KEGG) signaling pathway analysis

FLT3LG was used to perform KEGG analysis and GSEA in 33 tumors. KEGG is a comprehensive database that integrates genomic, chemical and system function information. It has 4 major categories and 17 subdatabases. Among them, GO is a database of KEGG that was established by the Gene Ontology Consortium, which aims to establish definitions and describe gene and protein functions applicable to various species. GO annotations are divided into three categories, molecular function (MF), biological process (BP), and cellular components (CC), which are used to define and describe the function of a gene in many aspects. The Database for Annotation, Visualization, and Integrated Discovery (DAVID) website (Version 6.8; https://david.ncifcrf.gov) was used to determine the KEGG signaling pathways and transcription factors associated with FLT3LG expression.

### qPCR

Total RNA was extracted using TRIzol reagent following the manufacturer's protocol. cDNA was synthesized using the PrimeScript RT Reagent Kit (TaKaRa). Quantitative PCR (qPCR) was performed using SYBR Green Master mix (Roche). The relative expression of genes was determined by the 2^-ΔΔCt^ method 28 and normalized to mActin expression levels. The gene-specific PCR primers (5' -3') are listed below:

*Cd25,* F: GCGTTGCTTAGGAAACTCCTGG; R: GCATAGACTGTGTTGGCTTCTGC;

*Cd69,* F: GGGCTGTGTTAATAGTGGTCCTC; R: CTTGCAGGTAGCAACATGGTGG;

*Flt3l*, F: GCCTGGAGCCCAAATTCCTC; R: GCTGAAGTAACAGTCAGGTGTC;

mActin: F: CGTGAAAAGATGACCCAGATCA; R: CACAGCCTGGATGGCTACGT;

### Flow cytometry

Single-cell suspensions prepared from tumor or spleen tissues were washed and stained for cell surface phenotyping using the following monoclonal antibodies: CD135 (FLT3) monoclonal antibody-APC (BV10A4H2) (eBioscience, 17-1357-42), CD8a monoclonal antibody-PE-Cyanine7 (RPA-T8) (eBioscience, 25-0088-42), and CD45 monoclonal antibody-eFluor™ 450 (HI30) (eBioscience, 48-0459-42). Pacific Blue anti-mouse CD11c (Biolegend, 117322) and PE anti-mouse CD4 (Biolegend, 100407) were used. For staining, 100 μL of single-cell suspensions was incubated with antibodies for 30 min, and individual single-color controls were prepared for compensation adjustment. Samples were washed with PBS and resuspended in 200 μL of PBS. Mouse IgG1 kappa Isotype Control-APC (P3.6.2.8.1) (eBioscience, 17-4714-82) was used as a isotype for FLT3 staining. Flow cytometry data were acquired using CytoFLEX LX (Beckman Coulter). Flowjo 10.0 was used for data analysis. An example of the gating strategy is given in **Supplementary Figure 1.**

### Statistical analysis

Data were analyzed by Prism 9.0 software (GraphPad). Kaplan-Meier and Cox analyses were used for survival analysis. Unpaired Student's *t* test, one-way ANOVA was used to analyze the significance of differences between groups. Data are presented as the mean ± standard deviation of three independent experimental repeats. P values <0.05 were considered statistically significant. ****P < 0. 0001, ***P < 0. 001; **P < 0. 01; *P < 0.05.

## Results

### Result 1 Elevated levels of FLT3LG induced by BCG immunotherapy

**Figure 1:**
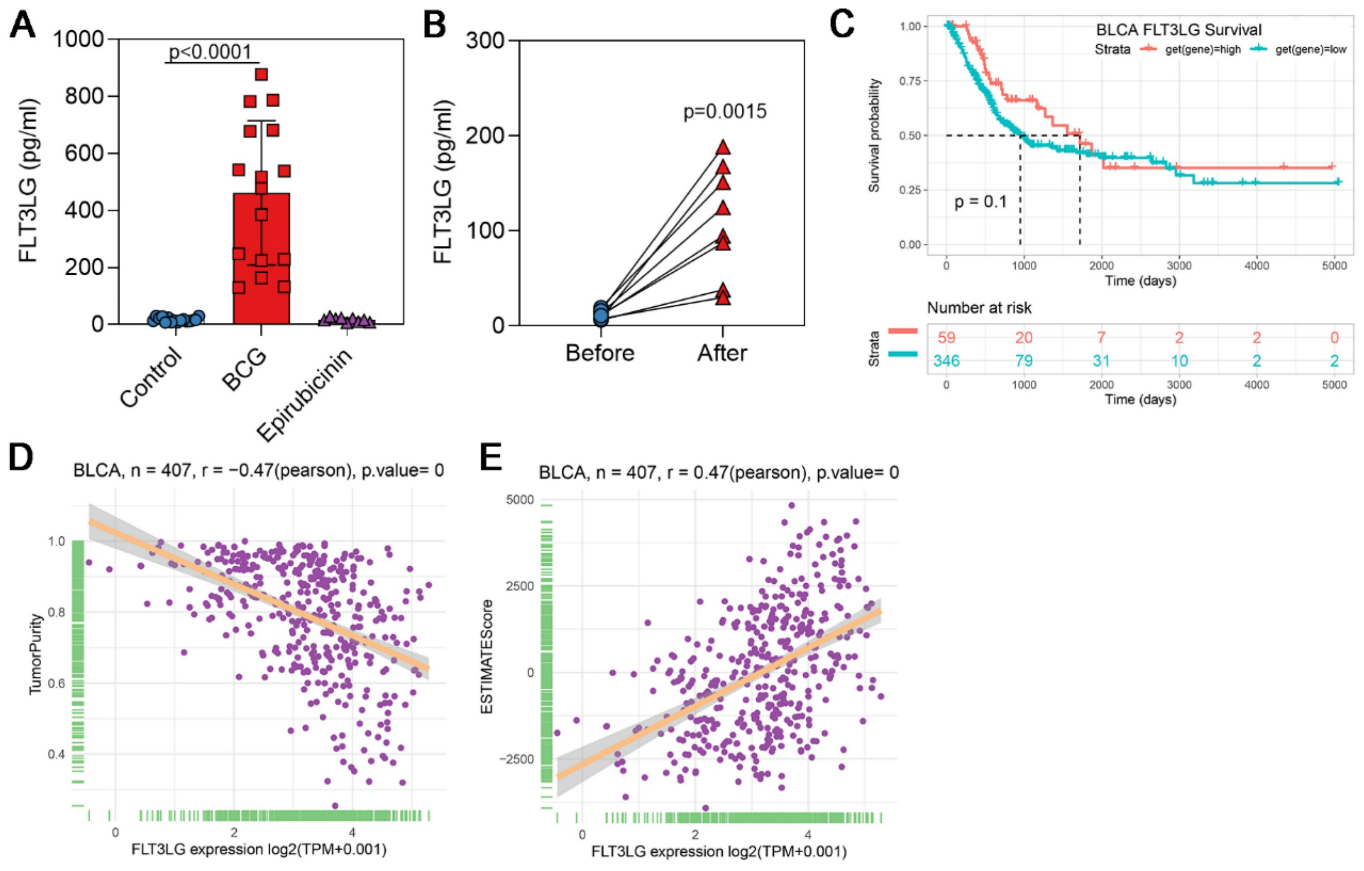
** FLT3LG levels were negatively correlated with tumor purity in BLCA. (A)** FLT3LG levels in urine samples from healthy individuals and patients before and after BCG instillation or epirubicin instillation were detected using the ELISA method;** (B)** Comparison of FLT3LG levels in urine samples from patients before and after BCG instillation;** (C)** In OS analysis, BLCA positively correlated with FLT3LG level. The correlation between FLT3LG and tumor purity **(D)** and ESTIMATE score **(E)**.

We first analyzed the levels of FLT3LG in urine samples from different instillation sources. The results showed that in the BCG group, the levels of FLT3LG (mean = 102.81 pg/ml) were approximately 6-fold higher than those in the epirubicin group (16.44 pg/ml) and the control group (16.94 pg/ml) **(Figure 1A)**, with significant differences. Comparing the urine samples from the same patient before and after instillation, a significant increase in FLT3LG levels could also be observed **(Figure 1B)**. We hypothesize that this significant elevation of FLT3LG is involved in the antitumor effects of BCG. Survival analysis of FLT3LG in BLCA showed a positive correlation with patient survival (p = 0.1) **(Figure 1C)**. Furthermore, we predicted the source of FLT3LG. Bioinformatics analysis revealed that the transcription level of FLT3LG was negatively correlated with the tumor purity of BLCA during tumor development** (Figure 1D)**. ESTIMATE analysis of FLT3LG also indicated a positive correlation not only with immune cells but also with stromal cells **(Figure 1E)**.

### Result 2 FLT3LG primarily participates in the activation of T cells in bladder cancer

**Figure 2:**
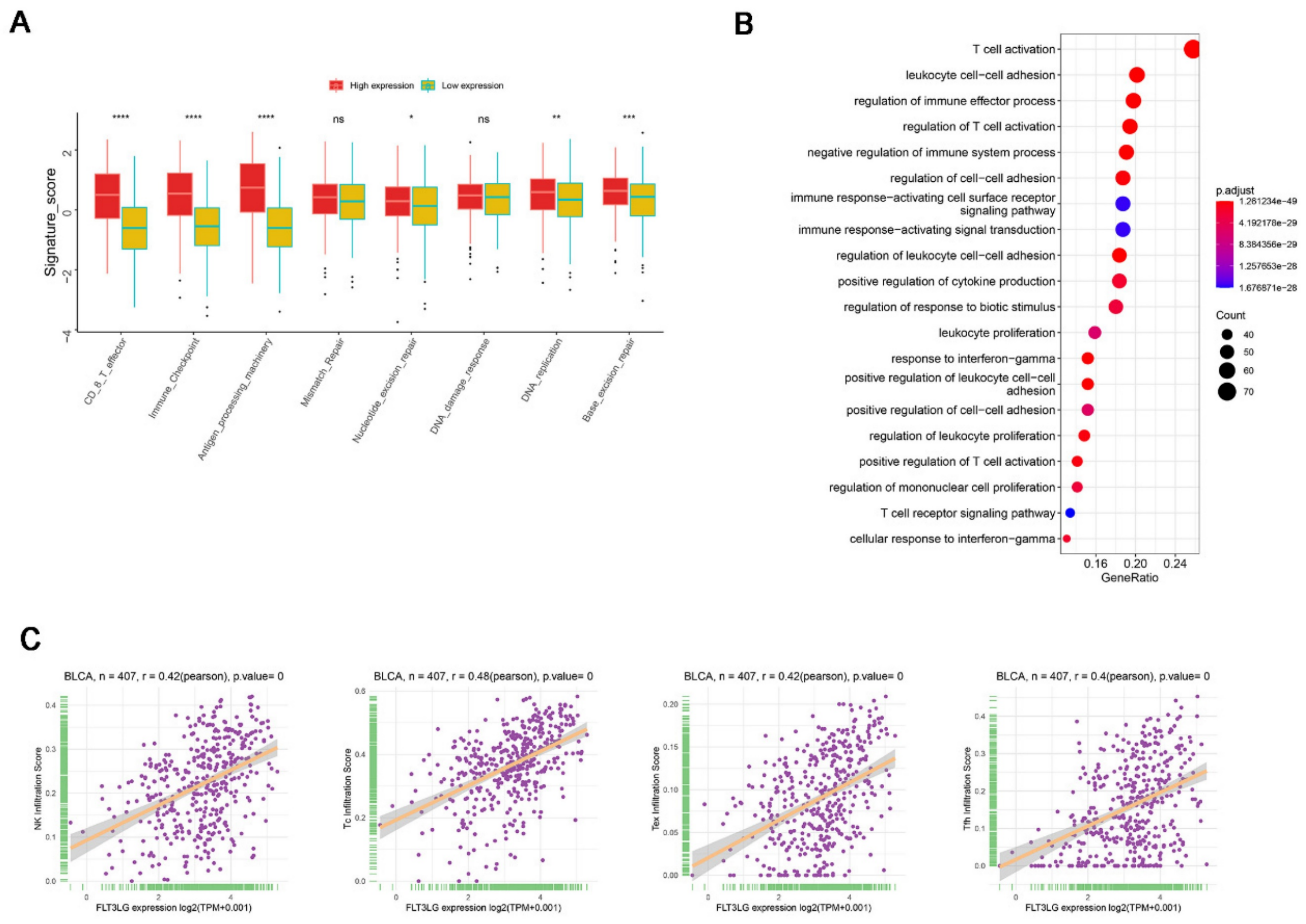
** FLT3LG regulates immune activation signals and is positively correlated with effector T cells. (A)** Correlation analysis between FLT3LG and the tumor microenvironment (TME). In BLCA, FLT3LG was positively correlated with immune checkpoints and CD8^+^ T effector cells. **(B)** GO_BP analysis of BLCA. The size of the circle represents the number of genes associated with the process, whereas the color represents the P value. **(C)** Correlation analysis of FLT3LG levels with 16 different immune cells and R≥0.4, p≤0.05 is shown.

The information above suggests that FLT3LG is likely involved in the regulation of the tumor immune response in BLCA. Therefore, we further analyzed the biological processes in which FLT3LG is involved in the bladder cancer microenvironment. In the analysis of FLT3LG and the tumor microenvironment, FLT3LG showed positive correlations with immune checkpoints, CD8^+^ T-effector cells, and antigen processing machinery in BLCA **(Figure 2A)**. Enrichment analysis of gene ontology biological processes in bladder cancer also indicated that FLT3LG is involved in T-cell activation, leukocyte cell‒cell adhesion, and regulation of the immune effector process **(Figure 2B)**. Due to the high correlation between FLT3LG and T-cell activation, we further analyzed the correlation between FLT3LG levels and each immune cell subtype using the ImmunCellAl database. In FLT3LG and immune infiltration correlation analysis, NK cells, cytotoxic T cells (Tc), central memory T cells (Tcm), follicular helper T cells (Tfh), and exhausted T cells (Tex) displayed a significant positive correlation with FLT3LG (R≥0.4, p≤0.05) (**Figure 2C**).

### Result 3 Activation of T cells mediated by FLT3LG in a mouse bladder cancer model

**Figure 3:**
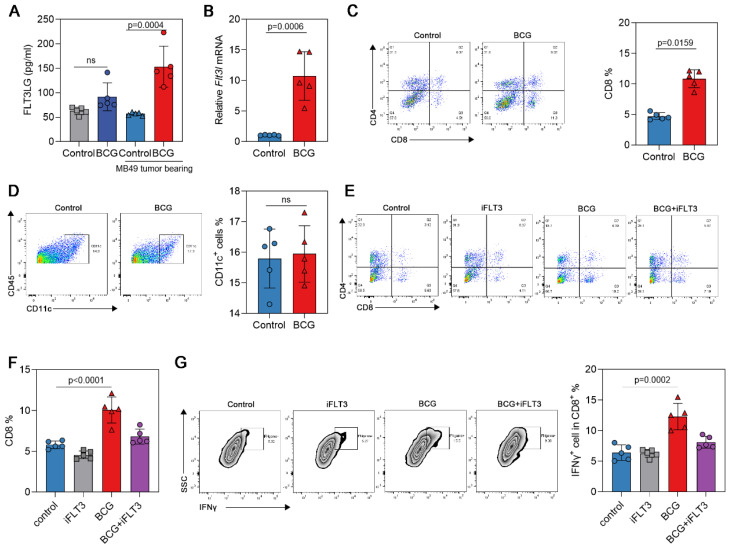
** FLT3LG was positively correlated with CD8^+^ T-cell activation in the MB49 tumor model. (A)** BCG was administered to tumor-bearing and nontumor-bearing mice, and FLT3LG levels in peripheral blood were measured. **(B)** On the second day after BCG injection, tumor tissues were isolated, RNA was extracted, and the transcription levels of FLT3LG within the tumors were detected. **(C)** Changes in the proportion of CD8^+^ T cells after BCG treatment. A representative flow cytometry figure (left) and statistical data (right) are shown. **(D)** Changes in the proportion of DC cells after BCG treatment. Representative flow cytometry plots (left) and statistical data (right) are shown. **(E)** Gilteritinib (30 mg/kg) via oral gavage was used to neutralize FLT3 pathway activation in the BCG treatment process. After 5 days of gilteritinib treatment, the CD8^+^ T-cell ratio was detected **(F)**, as well as the activation of CD8^+^ T cells **(G)**.

The bioinformatics analysis above revealed that FLT3LG is involved in T-cell activation during the development of bladder cancer and is positively correlated with several effector T-cell subtypes. Therefore, we further analyzed the role of FLT3LG in T-cell activation during BCG treatment in the MB49 bladder tumor-bearing mouse model. We established an MB49 bladder cancer subcutaneous tumor model in mice and administered BCG via peritumoral injection. The results showed a significant increase in peripheral blood FLT3LG levels compared to the control group on the second day after injection **(Figure 3A)**. However, it is worth noting that BCG treatment also resulted in a moderate increase in FLT3LG levels in peripheral blood compared to nontumor-bearing mice (BCG intravenous injection) (p=0.0848). This suggests that BCG may induce a systemic immune response. On the second day after BCG treatment, we collected tumor tissues and analyzed the changes in FLT3LG levels and tumor immune activation levels. Transcriptional levels of FLT3LG were significantly increased within the tumor tissue **(Figure 3B)**. The proportion of CD8 T cells within the tumor showed a significant increase **(Figure 3C)**, while the proportion of DC cells did not show significant changes **(Figure 3D).** Gilteritinib, a FLT3 inhibitor, has been extensively studied in acute myeloid leukemia but has rarely been used in mouse tumors. FLT3 is highly conserved between the human and mouse genomes^14^. There have also been reports on the use of FLT3L to promote the proliferation of DCs^15^, suggesting similar specificities in mice compared to humans. Therefore, in our study, we attempted to use gilteritinib to neutralize the FLT3 pathway activation induced during BCG treatment. After neutralizing FLT3LG function with the FLT3 inhibitor, there was a significant inhibition of the CD8^+^ T-cell ratio in tumors **(Figure 3E, F)**, as well as the activation levels of CD8^+^ T cells **(Figure 3G)**.

### Result 4 FLT3LG can synergize with the MHC-II pathway to promote TIL activation

**Figure 4:**
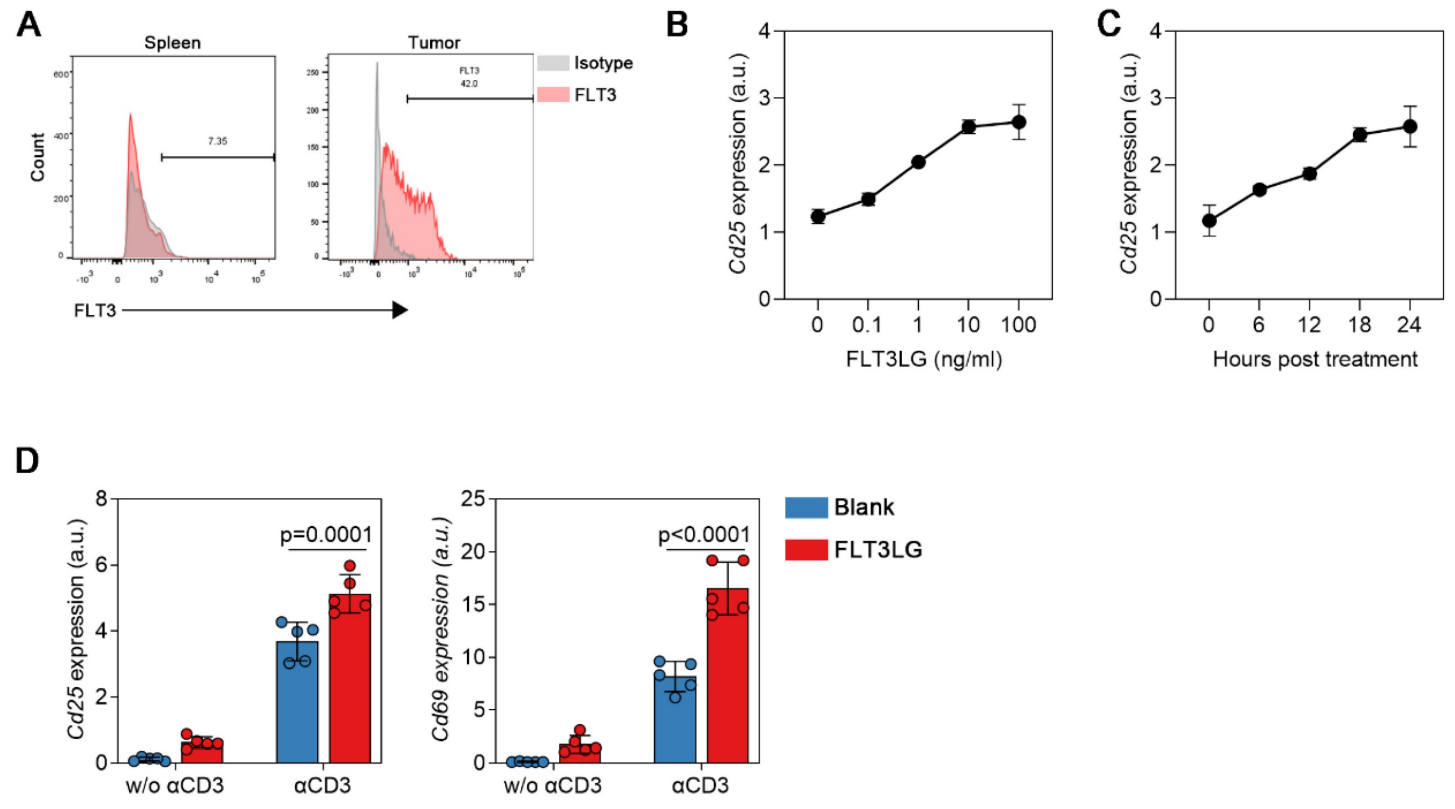
** FLT3LG synergistically activates tumor-derived CD8+ T cells with TCR activators. (A)** Typical flow cytometry figure of spleen or tumor-derived CD8^+^ T-cell FLT3 phenotype, isotype indicated isotype control of anti-FLT3 antibody;** (B)** MB49 tumor-derived CD8^+^ T cells treated with indicated concentration of mouse-FLT3LG for 24 hours, *Cd25* transcription level was measured using qPCR, n=3; **(C)** *Cd25* transcription level was analysis after 10 ng/ml FLT3LG treated in indicated time, n=3; **(D)** *Cd25* and* Cd69* transcription level was measured using qPCR after αCD3 and FLT3LG cotreatment, n = 5, data are presented as the mean ± SD.

To elucidate the signaling pathway of T-cell activation by FLTL3G, we analyzed the correlation between FLT3LG and immune-related genes in bladder cancer, selecting genes with R≥0.5 and p≤0.05, as shown in **Supplementary Table 1**. The results showed that in bladder cancer, FLT3LG not only exhibited significant positive correlations with immune checkpoints (CTLA4^16^, IDO1^17^), cytokines and chemokines (TNFSF13B, TNFRSF14, CXCR3, CCL4) but also exhibited the closest association with genes related to HLA^18^. This included HLA class I (HLA-A, HLA-B, HLA-C, HLA-E, HLA-F)^19^ and HLA class II (HLA-DP, HLA-DM, HLA-DO, HLA-DR)^20^. This suggests that FLT3LG is involved in antigen presentation and target cell recognition processes. Previous reports have shown that FLT3LG can promote T-cell activation by enhancing DC proliferation and antigen presentation^21^. Thus, we further identified the function of FLT3LG in direct T-cell activation. We sorted tumor-infiltrating T cells using flow cytometry and analyzed the levels of FLT3LG receptors on the surface of tumor-derived CD8^+^ T cells. FMS-like tyrosine kinase 3 (FLT3) is a receptor tyrosine kinase involved in hematopoietic cell proliferation, differentiation, and apoptosis 22. FLT3 further activates the phosphatidylinositol 3-kinase (PI3K) and RAS signaling pathways after being activated by its ligand FLT3LG (FL/FLT3LG/FLT3L). In the MB49 tumor model, flow cytometry analysis revealed a higher proportion of FLT3^+^ phenotype CD8^+^ T cells in tumor-derived CD8^+^ T cells compared to spleen-derived CD8^+^ T cells **(Figure 4A)**. This information suggests that tumor-derived CD8+ T cells can be directly activated by FLT3LG. In vitro, we used CD3 antibodies in conjunction with FLT3LG to stimulate tumor-derived CD8^+^ T cells, *Cd25* and *Cd69* was used for the marker as T cell activation^23^, ^24^. Tumor-derived CD8^+^ T cells were sorted and treated with FLT3LG, CD3 antibody (αCD3), or their combination to analyze the phenotypic changes in T cells. The results showed that 10 ng/ml FLT3LG activated tumor-derived CD8^+^ T cells **(Figure 4B)**, leading to an increase in *Cd25* transcription levels after 18 hours of stimulation **(Figure 4C)**. However, the increase was relatively small. Furthermore, we investigated whether FLT3LG could have a synergistic effect with a T cell receptor (TCR) activator. It was observed that the FLT3LG/FLT3LG pathway had a synergistic effect with αCD3 **(Figure 4D)**. With the addition of FLT3LG, αCD3 could significantly activate T cells. These findings suggest that FLT3LG can directly activate CD8^+^ cytotoxic T cells, indicating that BCG can promote the secretion of FLT3LG to directly activate CD8^+^ cytotoxic T cells, further enhancing its antitumor capabilities.

## Discussion

FLT3LG has been previously explored as an antitumor agent in solid tumor therapy^25^. Researchers hypothesized that FLT3LG might induce a powerful antitumor immune response^26^. Many studies on the antitumor potential of FLT3/FLT3LG have shown that FLT3LG, as a single agent, could delay or reverse the growth of methyl choline-induced fibrosarcoma^27^, C3L5 breast tumors^28^, B16 melanoma and EL4 thymoma (THYM) 29. Using tumor cell lines stably expressing FLT3LG, it has been confirmed that the antitumor effect of FLT3LG in these cases is associated with the expansion of DCs in lymphoid and peripheral tissues and activation of tumor antigen-specific T cells^25^. Some experiments have indicated that overexpression of FLT3LG can induce the expansion of tumor-infiltrating DCs^30^, ^31^. Studies from some research groups have shown that FLT3LG can exert antitumor activity, and there are reports that FLT3/FLT3LG can promote dendritic cell-mediated immune responses against malignant tumors^27^, ^28^, ^32^-^34^. In this study, our results demonstrate high levels of FLT3LG expression after BCG treatment in both human and mouse models** (Figure 1A, Figure 3A)**. However, our results show that, along with the increase in FLT3LG, the levels of DCs did not significantly increase during BCG treatment **(Figure 3D)**, which may be due to tumor heterogeneity.

It is also known that CD8^+^ T cells are the terminal effector cells in FLT3/FLT3LG antitumor effects^35^. Our results show that FLT3 is highly expressed in CD8^+^ T cells in the bladder cancer tumor model** (Figure 4A)**. This suggests that FLT3LG can directly activate T cells. Consistent with this result, FLT3LG significantly stimulated T-cell activation in vitro **(Figure 4D)**.

FLT3LG can be expressed in a variety of cell types, including stromal bone marrow cells and thymocytes^36^, ^37^, activated T lymphocytes^38^, and NK cells^39^. NK cells and other lymphocytes are considered to be important sources of FLT3LG in the TME^39^. Our results also indicate that during BLCA development, there is a significant positive correlation between NK cells and FLT3LG** (Figure 2C)**, which also suggests that NK cells may be a potential source of FLT3LG during BCG treatment.

Under physiological conditions, FLT3 is mainly expressed in dendritic cells (DCs) and lymphocyte precursors^40^ and promotes the proliferation and differentiation of T cell precursors^36^, ^41^. The activation of FLT3 by FLT3LG can also promote the activation of precursor cells^42^. FLT3/FLT3LG can promote monocyte differentiation in the absence of other growth factors^42^. In terms of tumor therapy, some studies have shown that overexpression of FLT3LG can induce the expansion of tumor-infiltrating DCs^30^, ^31^; FLT3/FLT3LG enhanced the immune response of DCs to malignant tumors by promoting their differentiation^27^, ^28^. Clinical experiments have demonstrated that FLT3LG can increase the number of DCs *in vivo* and increase the proportion of tumor antigen specific CD8^+^ T cells^43^.

In addition to FLT3LG, various cytokines, including IL-1β, IL-8, IL-15, IL-18, chemokine ligand 2 (CCL2), and CCL3, can be detected in the urine of BCG-instilled patients^44^. Bioinformatics analysis of FLT3LG and immune-related genes showed a significant positive correlation between FLT3LG and cytokines such as TNF, CXCR3, and CCL4 **Supplementary Table 1.** In previous studies, the FLT3/FLT3LG pathway was found to synergize with IL-3, G-CSF, GM-CSF, and EPO to stimulate a significant cell proliferation effect^45^. FLT3L, KIT ligand, and IL-3 together can have a synergistic effect to promote the proliferation of MDSCs^42^. FLT3LG can cooperate with IL-7 and IL-11 to promote hematopoietic progenitor differentiation and long-term cloning^46^. These previous results are partially consistent with our analysis of FLT3/FLT3LG function. Therefore, further confirmation of the synergistic interaction between FLT3LG and cytokines is needed in subsequent studies.

Previous literature has reported that BCG can enhance the function of MHC II antigen presentation 47. These findings are highly consistent with the bioinformatics analysis of FLT3LG and immune-related genes **Supplementary Table 1**. It is worth noting that BCG can also induce the expression of major histocompatibility complex class II (MHC-II) on urothelial cells^48^. This also suggests that FLT3LG may be involved in the antigen presentation function of urothelial cells. Further research can investigate the impact of FLT3LG on the function of urothelial cells in subsequent studies. In previous studies on the immunological mechanisms of BCG bladder instillation, researchers focused more on the classical CD8 T cell activation pathways and mechanisms^49^. In this study, we observed that BCG promotes an increase in local FLT3LG levels, directly activating cytotoxic CD8 T cells. This expands researchers' understanding of the cytokine effects and T cell activation in BCG immunotherapy, and provides a new perspective for optimizing strategies for BCG immunotherapy in the future.

## Supplementary Material

Supplementary figure and table.
