# Supplementary material for: BCG immunotherapy promotes tumor-derived T-cell activation through the FLT3/FLT3LG pathway in bladder cancer
Source: J Cancer. 2024 Jan 1;15(3):623–31. doi: 10.7150/jca.90085 (PMC10777044; doi:10.7150/jca.90085)
Supplement: Supplementary file 1 — Supplementary figure and table. [file jcav15p0623s1.pdf]

Supplementary Material

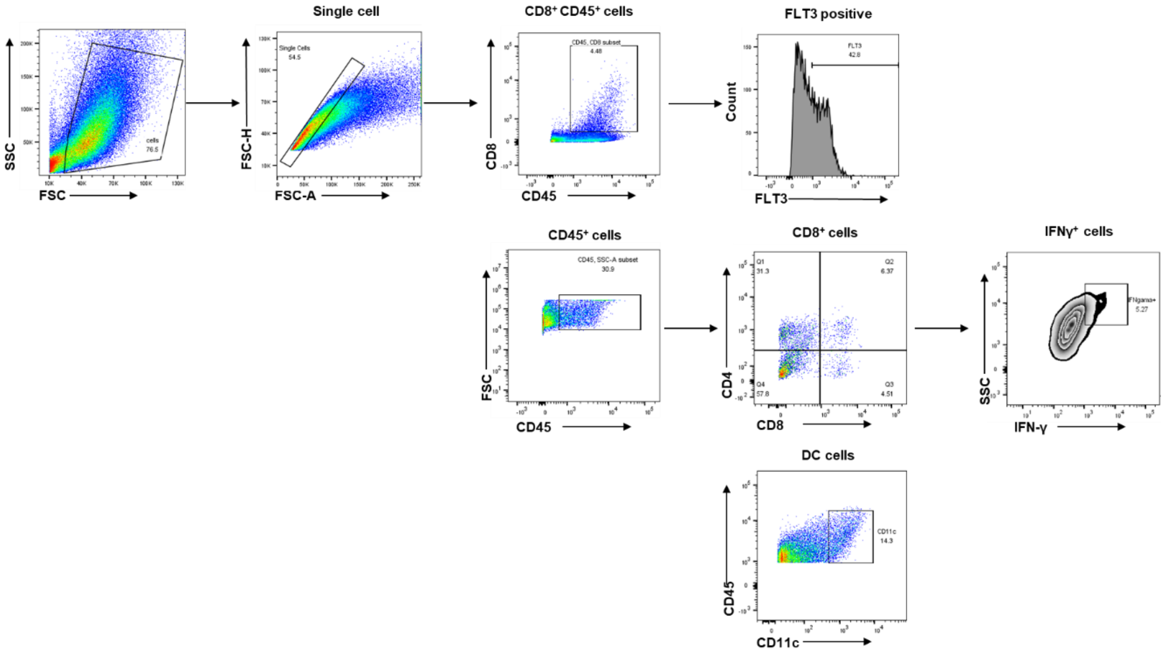

Supplementary Figure 1. Exemplifying gating strategies for FACS analysis are shown. Gating strategy for FLT3 level in tumor-infiltrating $CD8^+$ T cells (**Figure 4A**), $CD8^+$ T cell (**Figure 3C,E**),, DC cell(**Figure 4D**), and IFN-γ cell(**Figure 4G**).

Gene	correlation	pvalue	type
FLT3LG	1	0	BLCA
HLA-E	0.626258468	1.03E-45	BLCA
HLA-F	0.616696619	5.26E-44	BLCA
HLA-A	0.596339674	1.48E-40	BLCA
HLA-B	0.585269291	8.85E-39	BLCA
TAPBP	0.58366602	1.58E-38	BLCA
HLA-DMA	0.582507985	2.40E-38	BLCA
B2M	0.581757495	3.13E-38	BLCA
STING1	0.567427885	4.67E-36	BLCA
HLA-DPB1	0.564090946	1.45E-35	BLCA
KLRK1	0.561848962	3.07E-35	BLCA
HLA-DPA1	0.557919335	1.13E-34	BLCA
TNFSF13B	0.557578188	1.27E-34	BLCA
TNFRSF14	0.550557515	1.24E-33	BLCA
IDO1	0.545482862	6.28E-33	BLCA
HLA-DRA	0.544922411	7.50E-33	BLCA
HLA-DRB1	0.543886454	1.04E-32	BLCA
TAP2	0.542611685	1.55E-32	BLCA
HLA-DMB	0.53667623	9.78E-32	BLCA
LAG3	0.534337879	2.00E-31	BLCA
CXCR3	0.5291535	9.57E-31	BLCA
PDCD1	0.528937838	1.02E-30	BLCA
TAP1	0.528220071	1.27E-30	BLCA
HLA-DOA	0.514879618	6.23E-29	BLCA
CCL4	0.511548099	1.60E-28	BLCA
CTLA4	0.510329475	2.26E-28	BLCA
CCR5	0.505145069	9.61E-28	BLCA
HAVCR2	0.49881734	5.43E-27	BLCA
CXCL11	0.495925663	1.19E-26	BLCA
CD48	0.495444537	1.35E-26	BLCA
CD27	0.495408145	1.36E-26	BLCA
CSF1R	0.487467669	1.11E-25	BLCA
CXCL10	0.485676559	1.77E-25	BLCA
LGALS9	0.481028916	5.86E-25	BLCA
CCL5	0.480708457	6.36E-25	BLCA
CD86	0.478315777	1.17E-24	BLCA
HLA-DQA1	0.473601361	3.82E-24	BLCA
HLA-DOB	0.473549999	3.87E-24	BLCA
ICOS	0.467404106	1.76E-23	BLCA
CXCL9	0.464770332	3.35E-23	BLCA
PDCD1LG2	0.462733293	5.47E-23	BLCA
HLA-DQB1	0.460101413	1.03E-22	BLCA

LTA	0.455879076	2.79E-22	BLCA
TNFRSF9	0.451319276	8.10E-22	BLCA
IL2RA	0.450342512	1.02E-21	BLCA
CXCR6	0.450258864	1.04E-21	BLCA
CXCL16	0.446931888	2.22E-21	BLCA
KLRC1	0.443174982	5.21E-21	BLCA
TIGIT	0.440066166	1.05E-20	BLCA
HLA-C	0.434479579	3.60E-20	BLCA
CD40	0.431729357	6.57E-20	BLCA
TNFRSF4	0.426550208	2.00E-19	BLCA
CXCL13	0.423841242	3.56E-19	BLCA
CD274	0.420329221	7.46E-19	BLCA
VSIR	0.420118359	7.79E-19	BLCA
CCR6	0.41987988	8.19E-19	BLCA
XCL2	0.415105299	2.20E-18	BLCA
CCL3	0.414375457	2.56E-18	BLCA
CD28	0.409326129	7.14E-18	BLCA
CD80	0.404547119	1.86E-17	BLCA
TNFSF14	0.39990531	4.62E-17	BLCA
CD44	0.39461369	1.29E-16	BLCA
SAT1	0.393526814	1.59E-16	BLCA
KIR2DL3	0.389557634	3.37E-16	BLCA
CCR2	0.387623982	4.84E-16	BLCA
CD70	0.374113831	5.73E-15	BLCA
CCR1	0.373690249	6.19E-15	BLCA
HLA-DQA2	0.373529404	6.37E-15	BLCA
ADORA2A	0.372255588	7.99E-15	BLCA
CXCR5	0.372037732	8.30E-15	BLCA
CCL8	0.366014046	2.39E-14	BLCA
TNFSF13	0.357713574	9.91E-14	BLCA
CX3CL1	0.357329661	1.06E-13	BLCA
TNFRSF8	0.35520639	1.51E-13	BLCA
CD40LG	0.354016209	1.84E-13	BLCA
CCL2	0.353431501	2.03E-13	BLCA
BTLA	0.350421519	3.34E-13	BLCA
CCL7	0.349315783	4.01E-13	BLCA
CCL22	0.348263352	4.76E-13	BLCA
TGFB1	0.346869045	5.97E-13	BLCA
NT5E	0.342070869	1.29E-12	BLCA
CCL23	0.339900151	1.83E-12	BLCA
CCL13	0.338326866	2.34E-12	BLCA
TNFRSF18	0.334892793	4.01E-12	BLCA
CCL21	0.332518808	5.80E-12	BLCA
CCR4	0.330233208	8.24E-12	BLCA

CISD1	0.327990545	1.16E-11	BLCA
CCL19	0.326909601	1.37E-11	BLCA
CXCR4	0.321983674	2.86E-11	BLCA
KIR2DL1	0.316147131	6.73E-11	BLCA
FTH1	0.315755137	7.12E-11	BLCA
TNFSF4	0.314920799	8.04E-11	BLCA
TNFRSF13B	0.312957564	1.07E-10	BLCA
MICB	0.305382192	3.12E-10	BLCA
IL10	0.300237263	6.34E-10	BLCA
ENTPD1	0.298423648	8.12E-10	BLCA
DPP4	0.289939873	2.53E-09	BLCA
TNFRSF17	0.285819456	4.32E-09	BLCA
CCL18	0.276288005	1.45E-08	BLCA
CCL1	0.271475931	2.62E-08	BLCA
CD244	0.266741826	4.65E-08	BLCA
CRYAB	0.261128043	9.05E-08	BLCA
CXCL2	0.260657681	9.56E-08	BLCA
CCR8	0.260643379	9.58E-08	BLCA
CCL26	0.256163873	1.61E-07	BLCA
HLA-G	0.250915046	2.92E-07	BLCA
CXCL1	0.245966537	5.05E-07	BLCA
NECTIN2	0.24391808	6.32E-07	BLCA
CCL25	0.241638775	8.09E-07	BLCA
CD160	0.240121845	9.52E-07	BLCA
IL6	0.235447155	1.56E-06	BLCA
CXCL12	0.225947996	4.14E-06	BLCA
IL10RB	0.224528202	4.78E-06	BLCA
CCL24	0.219782124	7.64E-06	BLCA
WTAP	0.219610803	7.77E-06	BLCA
SLC1A5	0.211937603	1.62E-05	BLCA
CX3CR1	0.211906651	1.63E-05	BLCA
CXCL6	0.210526288	1.85E-05	BLCA
CCL17	0.209831591	1.97E-05	BLCA
CCL11	0.209027968	2.13E-05	BLCA
TMIGD2	0.201211018	4.34E-05	BLCA
CXCL3	0.199819807	4.91E-05	BLCA
ACO1	0.198623872	5.46E-05	BLCA
IL6R	0.192357502	9.41E-05	BLCA
CARS1	0.19010777	0.000113977	BLCA
ZEB1	0.188725282	0.000128064	BLCA
ICOSLG	0.186228442	0.000157733	BLCA
CXCL5	0.180123765	0.000259596	BLCA
MT1G	0.178630019	0.000292546	BLCA
KEAP1	0.173682898	0.000431657	BLCA

TGFBR1	0.171441757	0.000513099	BLCA
CCL14	0.169494088	0.000595239	BLCA
PTGS2	0.165892708	0.00078006	BLCA
G6PD	0.165136922	0.000825042	BLCA
RAET1E	0.163571404	0.000925938	BLCA
CCL20	0.162060027	0.001034039	BLCA
TNFRSF25	0.16057589	0.001151408	BLCA
PVR	0.160150581	0.001187237	BLCA
CXCR1	0.156937553	0.001492896	BLCA
HSPB1	0.156900352	0.001496824	BLCA
TNFSF18	0.148931787	0.002593953	BLCA
ALKBH5	0.148596347	0.002653191	BLCA
AIFM2	0.146983254	0.002955635	BLCA
GPX4	0.146522469	0.003047608	BLCA
CCR10	0.145951187	0.003165245	BLCA
CD276	0.144561405	0.003468832	BLCA
CXCL8	0.142347331	0.004007331	BLCA
PHKG2	0.140259729	0.00458318	BLCA
TNFRSF13C	0.138861613	0.005009535	BLCA
METTL14	0.132293253	0.007529665	BLCA
GSS	0.130702052	0.008289651	BLCA
CD96	0.112098321	0.023717439	BLCA
TNFSF9	0.10501279	0.034183836	BLCA
EMC2	0.099683409	0.044445962	BLCA
FTO	0.095389038	0.054496453	BLCA
ACSL4	0.086811163	0.080244961	BLCA
CCL28	0.082133352	0.097989733	BLCA
ACSL3	0.079665614	0.108537445	BLCA
NFE2L2	0.077975354	0.116264538	BLCA
CCR3	0.073559802	0.138484949	BLCA
RBM15	0.071494515	0.149936889	BLCA
CHAC1	0.046573085	0.348657995	BLCA
HNRNPC	0.044374274	0.371908659	BLCA
XCR1	0.044280706	0.372918859	BLCA
YTHDF1	0.043288672	0.383733131	BLCA
HHLA2	0.042081632	0.397146173	BLCA
CXCL17	0.038376686	0.440041112	BLCA
HMOX1	0.035936033	0.469688496	BLCA
ABCC1	0.035367333	0.476751369	BLCA
VIRMA	0.034513159	0.487467628	BLCA
NFS1	0.033448603	0.501002592	BLCA
TNFSF15	0.033370999	0.501996969	BLCA
NCOA4	0.032935828	0.507592209	BLCA
CXCR2	0.032425707	0.514192417	BLCA

CCL27	0.028581228	0.565326024	BLCA
GCLM	0.02434098	0.624400674	BLCA
CCR9	0.017885053	0.719044257	BLCA
STEAP3	0.01609996	0.746069395	BLCA
SLC7A11	0.005686646	0.908943024	BLCA
HSBP1	0.000390508	0.993733496	BLCA
CCL16	-0.005282271	0.915392897	BLCA
CS	-0.014791569	0.766079336	BLCA
YTHDC2	-0.015243874	0.759143632	BLCA
XCL1	-0.017346588	0.72716125	BLCA
ULBP1	-0.017744819	0.721155189	BLCA
GOT1	-0.019371	0.696810277	BLCA
CXCL14	-0.021721886	0.662164781	BLCA
RPL8	-0.025683687	0.605407127	BLCA
YTHDF2	-0.030356137	0.541418577	BLCA
VTCN1	-0.034122632	0.492409901	BLCA
TP53	-0.034277515	0.490446602	BLCA
YTHDC1	-0.039055724	0.431986947	BLCA
LPCAT3	-0.047606319	0.338057336	BLCA
FADS2	-0.047742407	0.336676654	BLCA
ALOX5	-0.050608004	0.308444821	BLCA
NOX1	-0.051043226	0.304297591	BLCA
ATP5MC3	-0.057076755	0.250607327	BLCA
NQO1	-0.059105513	0.234132137	BLCA
IREB2	-0.0670843	0.176780537	BLCA
CCR7	-0.068310567	0.168981924	BLCA
ACSF2	-0.075315813	0.129286538	BLCA
AKR1C1	-0.077455993	0.118723379	BLCA
ALOX12	-0.084669628	0.088016192	BLCA
PGD	-0.086802278	0.080276015	BLCA
ZC3H13	-0.095585635	0.053998245	BLCA
KDR	-0.101268053	0.041154042	BLCA
CBS	-0.103480092	0.036904701	BLCA
ACACA	-0.110147537	0.026278322	BLCA
ALOX15	-0.121535235	0.014149669	BLCA
BTNL2	-0.129855181	0.008721398	BLCA
FANCD2	-0.148737452	0.002628124	BLCA
METTL3	-0.163333791	0.000942211	BLCA
CCL15	-0.165057455	0.000829908	BLCA
TFRC	-0.167674573	0.000682837	BLCA
SQLE	-0.17132343	0.000517772	BLCA
GLS2	-0.180981454	0.000242278	BLCA
AKR1C3	-0.184250848	0.000185684	BLCA
AKR1C2	-0.188943256	0.000125739	BLCA

GCLC	-0.2190975	8.16E-06	BLCA
HMGCR	-0.220364711	7.21E-06	BLCA
FDFT1	-0.22597269	4.13E-06	BLCA
PEBP1	-0.301816844	5.11E-10	BLCA

Supplementary Table 1. Results of correlation analysis between FLT3LG and immune-related genes.
